# Formation of Anisotropic Flow-Induced Morphologies above the Equilibrium Melting Temperature in Isotactic Polypropylene

**DOI:** 10.1021/acs.macromol.6c00217

**Published:** 2026-04-27

**Authors:** Benson J. Jacob, Xiaoshi Zhang, Jongkyeong Kim, Kirt A. Page, Alicyn M. Rhoades, Ralph H. Colby

**Affiliations:** † Department of Chemical Engineering, The Pennsylvania State University, University Park, Pennsylvania 16802, United States; ‡ Department of Materials Science and Engineering, The Pennsylvania State University, University Park, Pennsylvania 16802, United States; § School of Engineering, Penn State Behrend, Erie, Pennsylvania 16563, United States; ∥ Cornell High Energy Synchrotron Source (CHESS), Cornell University, Ithaca, New York 14853, United States

## Abstract

Shearing an isotactic polypropylene melt, even above its equilibrium melting temperature (*T*_m_° ∼ 187 °C) leads to the formation of stable flow-induced precursors. Anisotropic structures result from shearing at temperatures as high as 220 °C (>30 °C above *T*_m_°), and are no longer generated at shearing temperatures exceeding 230 °C. The long periods increase with deformation, accompanied by a change in structural morphology. The crystallization kinetics were strongly affected by flow at shearing temperatures near *T*_m_° and persist at even higher shearing temperatures, as chains continue to be aligned and stretched with large deformations, perhaps further stabilized by stretched chains adsorbing onto heterogeneous impurities from polymerization.

## Graphical abstract

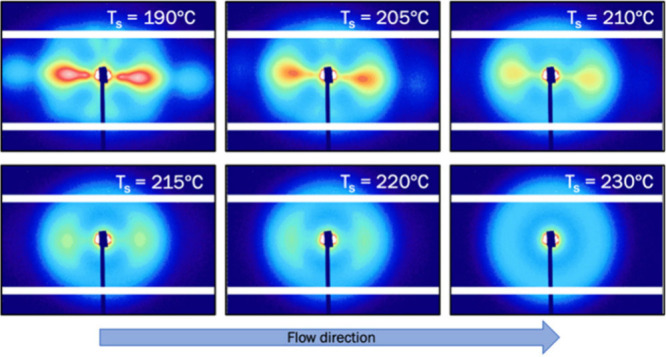

## Introduction

Distinct from their small-molecule counterparts, long chain polymers are slow crystallizers and only ever manage to be of order half crystalline. The slow nucleation of polymer crystals is known to be accelerated by the application of shear flows that stretch chains, resulting in a finer morphology with many tie-chains between crystals that make thermoplastics tough. The equilibrium melting temperature (*T*_m_°) is the thermodynamic limit of even the perfect crystal with no amorphous defects.^1–3^ Yet, we find shearing isotactic polypropylene (iPP) accelerates nucleation at temperatures as high as *T*_m_° + 30 °C, strongly supporting the hypothesis that stretched chains adsorb to particulate impurities from polymerization, which appear to impart enhanced stability.

In polymer processing, flow-induced crystallization is an important phenomenon, as early structure formation during flow is closely related to the final material properties of semicrystalline polymers.^4–6^ The most prominent change in structure formation associated with flow effects consists of a transformation in morphology from isotropic spherulites to anisotropic superstructures, known as *shish-kebabs*. These superstructures have been shown to improve tensile strength,^7^ decrease permeability,^4,8^ and increase stiffness^4,9^ of materials. Due to experimental complexities, the formation and systematic study of such structures at elevated shearing conditions have been evasive, limiting insights into practical processing conditions.

In polymer crystallization, the growth rate is expected to go to zero near the glass transition, while the nucleation rate goes to zero at the equilibrium melting temperature as the driving force for crystallization is dissipated.^10^ *T*_m_° is obtained by extrapolation of the Gibbs–Thomson melting line^11^ to an infinite lamellar thickness (melting temperature, *T*_m_, vs reciprocal lamellar thickness, *d*_c_^–1^) or approximated through the Hoffman–Weeks procedure,^3^ such that when the melting temperature vs crystallization temperature (*T*_c_) lines intersect (*T*_m_ = *T*_c_ at *T*_m_°). Both are presented in Figure 1 using literature data for iPP. For isotactic polypropylene, these two methods conclude that the equilibrium melting temperature is ∼187 °C.^1,11–16^

**Figure 1:**
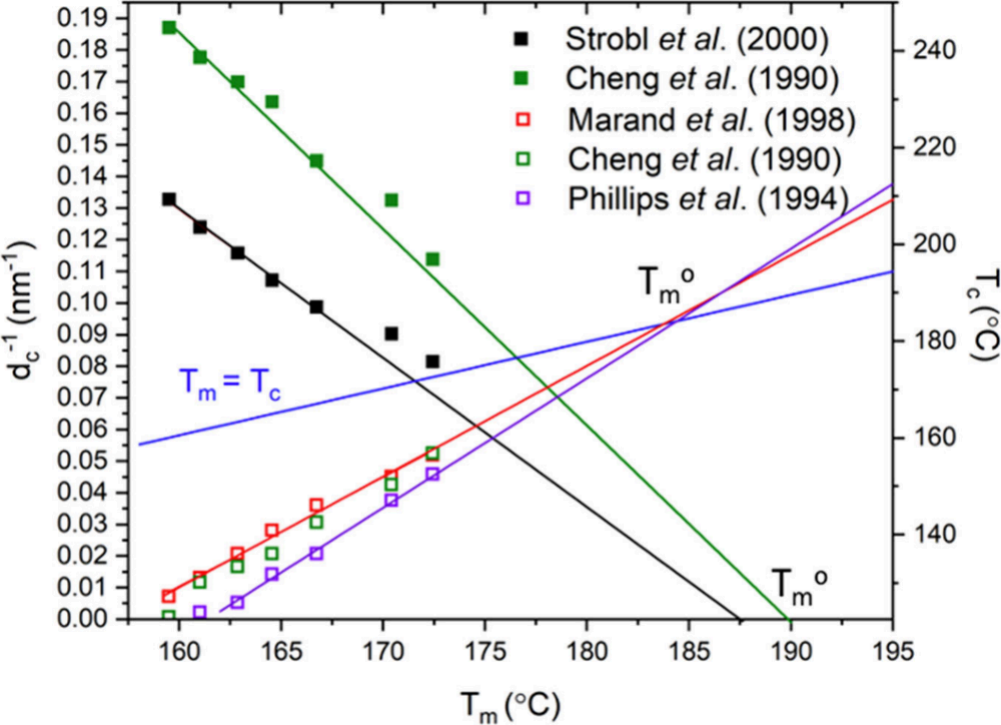
Determination of the equilibrium melting temperature for iPP using both the Gibbs–Thomson and linear Hoffman–Weeks methods. Both methods agree with one another, where the equilibrium melting temperature of iPP is ∼187 °C. Data have been extracted from refs 11, 14, 16, and 17 .

The equilibrium melting temperature is the thermodynamic limit of the perfect crystal with no amorphous defects. Data used to construct Figure 1 have been extracted from Strobl et al.,^11^ Marand et al.,^17^ Cheng et al.,^16^ and Phillips et al.,^14^ yielding similar results. Marand et al. have also determined a higher melting value of *T*_m_^XM^ = 212.1 °C, where a nonlinear fit was used for extrapolation to consider lamellar thickening during the slow increase in temperature. Similar observations have also been made by Phillips et al.^14^ who argued that thickening in polypropylene favors the thicker lamellae, causing an abnormally high value of the equilibrium melting temperature, and also concluded the equilibrium melting temperature of iPP is ∼186 °C. Cheng et al.^16^ obtained an equilibrium melting value ∼ 185 °C through the Gibbs–Thomson method, while in Figure 1, a slightly higher value of ∼190 °C is seen, as lower melting temperature data are not plotted. While more crystal-mobile polymers (HDPE and PEO) clearly do need a curving Hoffman–Weeks analysis, it is not at all clear that any curvature is necessary for iPP. The three Hoffman–Weeks data sets in Figure 1 are linear and agree reasonably with the Gibbs–Thomson results.

With the application of shear flow, polymer chains can be aligned and stretched, lowering their conformational entropy, which increases the free energy of the melt. This effectively decreases the nucleation barrier and can increase the number density of nuclei.^18–22^ It was thought that these stable clusters (dormant nuclei) can exist only at temperatures below the equilibrium melting temperature.^2,23^ As such, polymer melts are often sheared near, or even below, their nominal melting temperatures, generating strong crystallization effects, allowing for molecular mechanisms and the development of anisotropic structures to be more readily explored in complex flow fields.^24–27^ However, Balzano et al. demonstrated that it was possible to produce a suspension of *only* shish crystals when polyethylene was sheared just 0.8 °C above its equilibrium melting temperature.^28^ In contrast, Somani et al. sheared samples of polypropylene as high as 195 °C (∼8 °C above *T*_m_°) and were unable to induce any oriented structures with a shear rate of 60 s^–1^ for 5 s.^29^ The temperature at which a polymer melt is sheared plays an important role in the nucleation and relaxation of flow-induced precursors, yet the factors that drive the formation and stability of such structures remains unclear at temperatures above *T*_m_°. These flow-induced precursors are thought to be metastable domains of aligned and stretched polymer chains that exhibit an intermediate level of order between the amorphous melt and the crystalline state, from which crystal nuclei may develop that are “invisible to scattering techniques”.^30^

In this work, we present the formation of anisotropic precursors well above the equilibrium melting temperature of isotactic polypropylene, generated in the presence of shear flow. Dynamic rheology was used to probe changes in flow-induced nucleation kinetics, while *ex-situ* small-angle X-ray scattering (SAXS) was employed to characterize the structural and morphological developments after the application of high temperature shear. We demonstrate that anisotropic structures can be generated as high as 220 °C (>30 °C above *T*_m_°), while characteristically distinct morphologies are induced at each of the respective shearing profiles. Flow effects persist at even higher temperatures, as observed by the viscoelastic properties of the sample. The nucleation kinetics were found to be robust at temperatures close to the equilibrium melting temperature and were progressively reduced at temperatures well exceeding the equilibrium melting temperature. The work presented here will better guide the development of existing polymer processing techniques to create materials for new applications, aid in recycling efforts, and enhance the mechanical performance of materials without the use of additives.

## Experimental Section

The iPP sample used in this work has strictly linear chains, a weight-average molecular weight of *M*_w_ = 833 kg/mol, dispersity of *M*_w_/*M*_n_ = 11.4, and the high *M* tail of the molecular weight distribution has the form ∼exp­(−*M*/*M*_max_), with *M*_max_ = 7000 kg/mol. The nominal melting temperature of quiescently crystallized iPP is *T*_m_ ∼ 165 °C. The xylene-soluble fraction is 3.6%, indicative of atactic content. Additional material properties of this sample (denoted iPP6) and extensive experimental methods are also detailed elsewhere.^31–33^

Prior to X-ray scattering, samples were sheared using a strain-controlled ARES G2 rotational rheometer (TA Instruments, New Castle, DE), under nitrogen to minimize sample oxidation, using 25 mm parallel plates with a constant gap height of 0.9 mm and a 500 s^–1^ perimeter shear rate for 1 s. This short strong interval of shear was chosen to somewhat imitate processing relevant conditions encountered in extrusion, 3D printing, and injection molding.

The X-ray scattering experiments were performed at the Cornell High Energy Synchrotron Source (CHESS) at the Functional Materials Beamline.^34,35^ The SAXS patterns are shown as a function of the scattering wavevector, *q* = (4π/λ)­sin­(θ/2), where θ is the scattering angle and wavelength λ = 1.278 Å. The sample-to-detector distance is 2453.64 mm, where the beam size was 0.5 mm by 0.04 mm using an exposure time of 0.3 s. SAXS patterns were analyzed using Igor Pro (WaveMetrics, Portland, OR) with the Nika 2D SAS macros package.

All rheological measurements were carried out after shearing, using 8 mm parallel plates with a constant gap height of 0.9 mm. The viscoelastic response as a function of time or temperature was used to probe crystallization dynamics. In both types of oscillatory experiments, a constant frequency of 0.5 rad/s and strain amplitude of 0.05 was used. The crystallization temperature was determined as the temperature at which the loss tangent tan δ ≡ *G*′′/*G*′ = 1 using a cooling rate of 3 °C/min. The crystallization time was similarly determined as the time at 150 °C for *G*′ to cross *G*′′ (tan δ ≡ *G*′′/*G*′ = 1) after a ∼10 °C/min “quench” to *T*_c_ = 150 °C.

## Results and Discussion

### Anisotropic Structure Development above the Equilibrium Melting Temperature

Figure 2 shows *ex-situ* SAXS patterns sheared at different temperatures above the equilibrium melting temperature of isotactic polypropylene (*T*_m_° ∼ 187 °C)^1,11–14^ using a rotational rheometer equipped with 25 mm parallel plates (γ̇_perimeter_ = 500 s^–1^, and fixed shearing time *t*_s_ = 1 s), meaning that the shear rate is linear in radial position. Prior to shear, samples were equilibrated at 220 °C for 5 min to erase any thermal or shear history. After the interval of shear (at the respective shearing temperature), samples were immediately cooled to room temperature at 10 °C/min, which is significantly slower than the cooling rate in injection molding.

**Figure 2:**
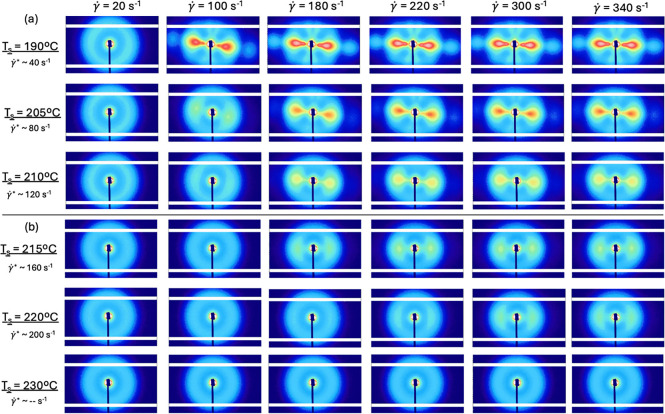
Small-angle X-ray scattering at room temperature after samples were sheared at their respective shearing temperatures using a perimeter shear rate of 500 s^–1^ for 1 s. (a) Structural evolution of shish-kebab structures in the shearing temperature regime of *T*_s_ = 190–210 °C. At these lower shearing temperatures, stronger anisotropic scattering is present at lower levels of deformation. (b) Structural evolution in the shearing temperature regime of *T*_s_ = 215–230 °C. Weaker shish-kebab morphologies are formed at 215 and 220 °C, while only isotropic structures are present at 230 °C, regardless of the shear rate applied (≤500 s^–1^). The flow direction is horizontal.

The shear rates listed at the top of Figure 2 are at a particular radial position at each shearing temperature. Strong anisotropic scattering is seen *slightly* above the equilibrium melting temperature at *T*_s_ = 190 °C and monotonically decreases as the shearing temperature is increased. Remarkably, anisotropic lamellar crystals persist with shearing temperatures as high as 220 °C (>30° above *T*_m_°) at γ̇ > γ̇* = 200 s^–1^. Approximate critical shear rates γ̇* for such flow-induced crystallization effects at each shearing temperature, as determined later, shown in Figure 3, are stated on the left side of Figure 2.

**Figure 3:**
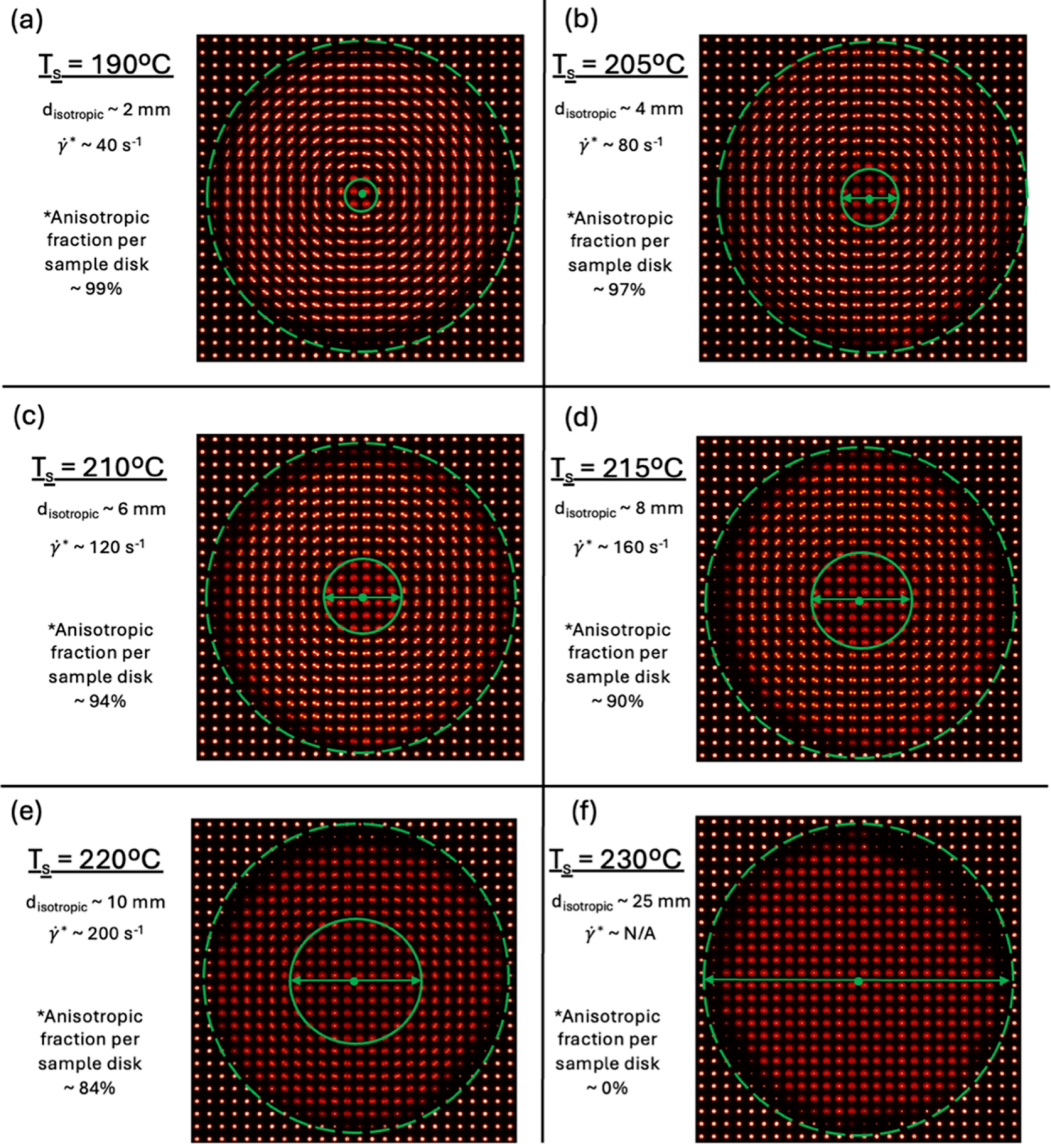
Ex-situ SAXS morphology evolution for six different shearing temperatures on a 25 mm diameter sample. Samples were sheared in 25 mm diameter parallel plate geometry using a perimeter shear rate of 500 s^–1^ for 1 s. The critical shear rate for anisotropic structure formation (indicated by the smaller green circle in each part) increases with elevated shearing temperatures. The fraction of anisotropic structures per sample disk is lowered with higher shearing temperatures and are no longer present at a shearing temperature of 230 °C.

However, upon shearing at *T*_s_ = 230 °C, anisotropic structures cease to form, regardless of shear rate (explored up to 500 s^–1^), suggesting these anisotropic precursors are no longer metastable at this temperature. Janeschitz-Kriegl et al. were able to use birefringence measurements at 210 °C to observe orientation and measure the relaxation times of precursors, where at temperatures above this, relaxations were too fast to measure.^22,36^ Similarly, at temperatures as high as 215 °C, Fernández-Ballester et al. indicated no growth in birefringence and the presence of large spherulites covering the entire sample upon cooling. It was suggested that shish structures could be induced at this temperature, but decay upon cessation of flow for temperatures exceeding 205 °C.^37^ Yet, no anisotropic structures could be observed at these elevated shearing temperatures.

At temperatures near *T*_m_°, the SAXS patterns show strong equatorial scattering corresponding to chain-extended shish structures,^5,28,31,38^ which weaken with elevated shearing temperatures. Consequently, the kebab structures, emanating radially from the shish, and oriented perpendicular to the flow direction, also exhibit reduced scattering with higher shearing temperatures, perhaps due to a smaller fraction of shish crystals to epitaxially nucleate from.^25,31,39^ Simulations have shown that even a single aligned chain (shish) can act as a “template” to form a row of crystal nuclei (kebabs) along the stretched chain.^40^

The SAXS data at every position on the 25 mm parallel plate sheared samples are shown in Figure 3 at six different shearing temperatures. One can easily see that the scattering is isotropic at lower shear rates (inside the smaller green circles) and anisotropic at larger shear rates, such that the green circles indicate the critical shear rate γ̇^∗^ at five of the six shearing temperatures (no anisotropic scattering was observed at *T*_s_ = 230 °C at any shear rate up to 500 s^–1^).

The critical shear rates for 1 s of shear flow (followed by a 10 °C/min “quench” to crystallize) to create anisotropic morphology in SAXS (Figures 2 and 3) are plotted in Figure 4, along with the relaxation time determined by fitting oscillatory shear complex viscosity data to the Cross Model

$$(\eta (\mathrm{\gamma ̇})\;=\;\eta_{o}/[1\,+\;(\mathrm{\gamma ̇}\tau {)}^{m})])$$

with shear thinning exponent, *m* = 0.7 for this polydisperse iPP.^33^

**Figure 4:**
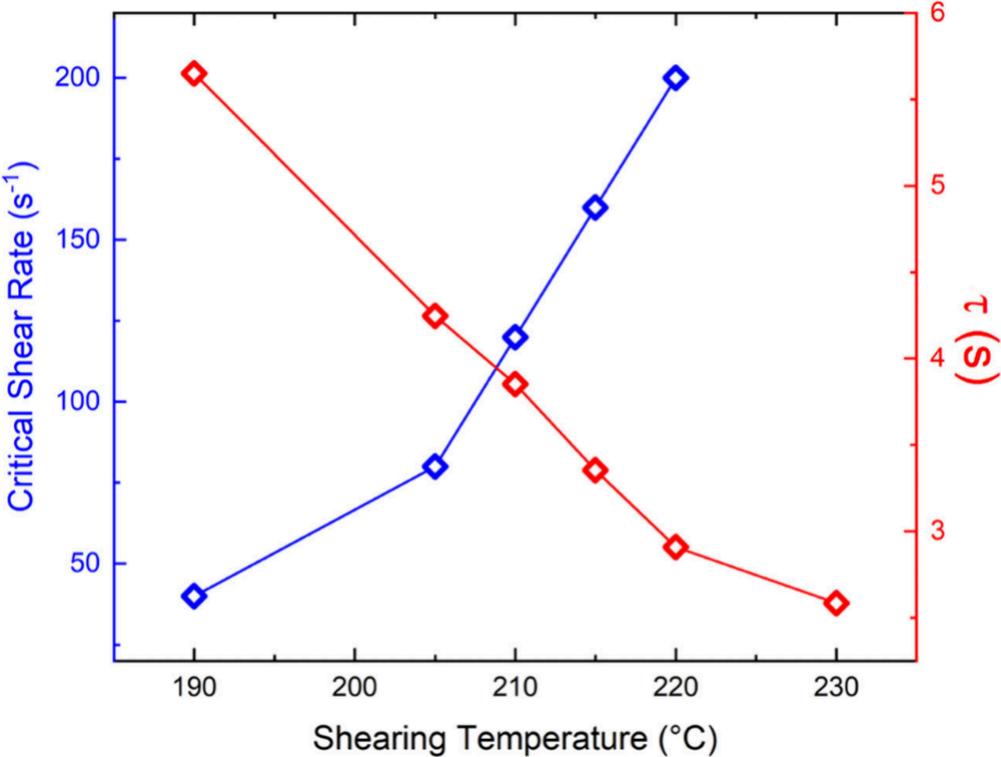
Shearing temperature dependences of critical shear rate obtained from the inner green circles in Figure 3. The relaxation time (τ) vs shearing temperature obtained by fitting complex viscosity to the Cross Model.

Figure 5 presents the *ex-situ* Lorentz-corrected (*Iq* ^2^) intensity profiles and long period development at increasing shearing temperature and deformation rates. As the shearing temperatures increase, the scattering intensity of the long period peak at *q* ∼ 0.03 Å^–1^ gradually weakens, and shifts toward higher wavevector values (see Figure 5a). For samples sheared at temperatures slightly above *T*_m_° (*T*_s_ = 190 °C), strong second order scattering peaks are present at *q* ∼ 0.075 Å^–1^, which no longer form at temperatures exceeding 210 °C. From the inset plot of Figure 5a, the maximum scattering intensity of the kebab structures exponentially decays with elevated shearing temperatures, where a significant decrease in scattering intensity is also seen at temperatures exceeding 210 °C.

**Figure 5:**
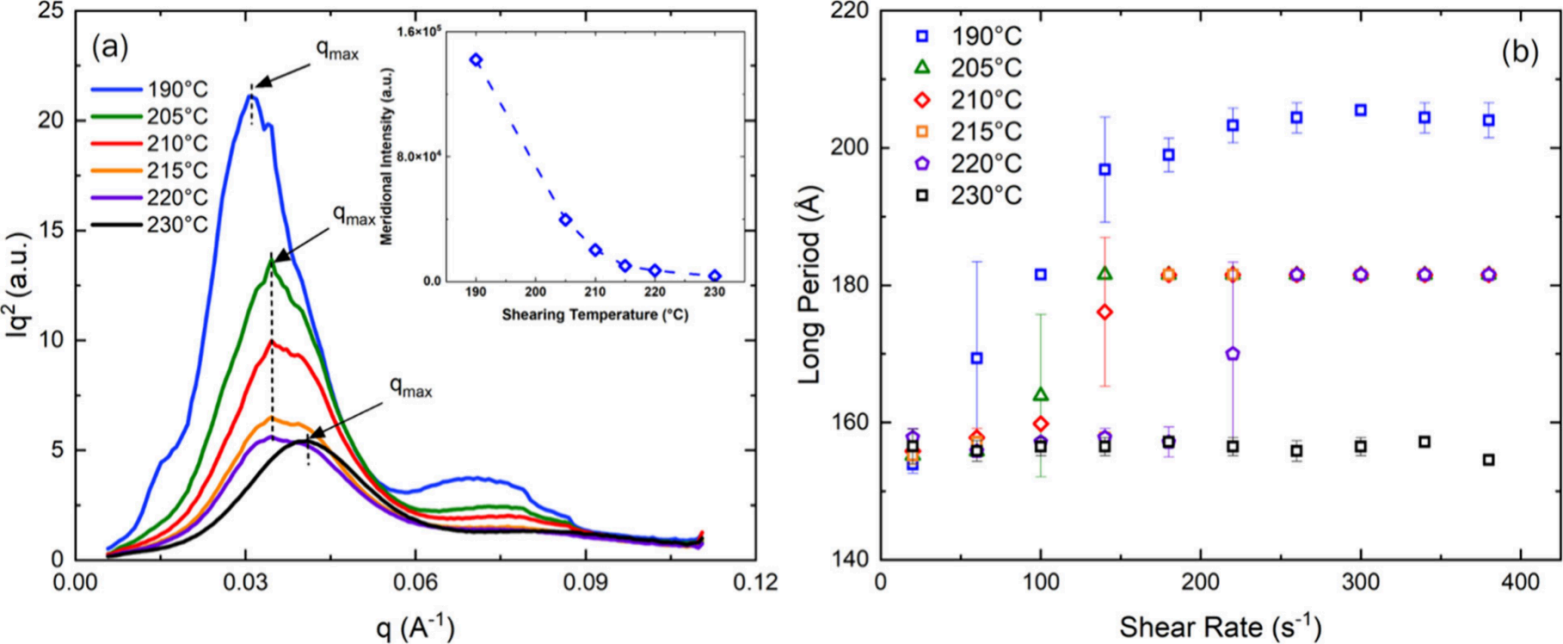
(a) Selected *ex-situ* SAXS intensity profiles at various shearing temperatures (*T*_s_ = 190–230 °C) using a constant shear rate of 300 s^–1^ for *t*_s_ = 1 s. The relative peak position is the same in the shearing temperature range of *T*_s_ = 205–220 °C. The inset shows the intensity of kebab structures, decreasing as a function of shearing temperature. (b) Shear rate evolution of lamellar spacing at different shearing temperatures using a perimeter shear rate of 500 s^–1^ for *t*_s_ = 1 s. The long period values are averaged over four points at the same radial position using 25 mm parallel plates, with the error bars indicating the standard deviation.

Interestingly, Marand et al. have proposed a higher *T*_m_° for iPP of ∼212 °C, utilizing a nonlinear Hoffman–Weeks extrapolation, taking into account the continuous thickening of lamellae during a slow increase in temperature,^17^ which appears to play an important role in accelerating the relaxation that reduces the orientation of these anisotropic structures for crystal-mobile polymers.^41^ Polyethylene and poly­(ethylene oxide) have no side groups and are found to be the most crystal-mobile polymers,^41^ each showing sizable curvature in their Hoffman–Weeks plots.^42,43^ Polypropylene has a methyl side group and is reported to be more than two orders of magnitude slower at rearranging its crystal,^41^ perhaps underlying the strong criticism of Marand’s use of a curving Hoffman–Weeks analysis for iPP.^11,12,44^

From Figure 5b, the long period, or spacing between adjacent crystalline lamellae can be determined through the SAXS intensity maxima such that *L*_p_ = 2π/*q*_max_ (with *q*_max_ indicated in Figure 5a) which is the sum of the average crystalline and amorphous layer thicknesses of the anisotropic kebab structures. For the shearing temperature of *T*_s_ = 190 °C, the long period grows rapidly as a function of deformation, and consistently exceeds that of all higher temperatures. Even at temperatures close to the equilibrium melting point, large deformations can still stretch and sustain chain orientation without being quickly relaxed back into the melt state. In the shearing temperature range of *T*_s_ = 205–220 °C, the initial long periods are gradually reduced with temperature, and exhibit a decay in their initial slopes at low levels deformation prior to reaching a plateau.

The initial long period values increase with a transition in morphology from isotropic structures to anisotropic morphologies at a critical shear rate, which increases with higher shearing temperatures (see Figure 3). For the shearing temperatures of *T*_s_ = 190, 205, 210, 215, and 220 °C, the respective critical shear rates for anisotropic structure development are ∼40 s^–1^, ∼80 s^–1^, ∼120 s^–1^, ∼160 s^–1^, and ∼200 s^–1^. At the highest shearing temperature of *T*_s_ = 230 °C, the long period remains constant and is independent of deformation, as anisotropic precursors relax rapidly and dissolve back into the melt state, perhaps as precursors formed at 230 °C are below a critical size to form a crystal lattice of shish structures.^28^

### Rheology: Flow-Induced Nucleation Kinetics

Figure 6 presents the time evolution of storage modulus *G*′ at 0.5 rad/s, after shearing at five different temperatures for 1 s at a perimeter shear rate of 500 s^–1^ and “quenching” at ∼10 °C/min to the crystallization temperature of 150 °C. The quiescent (not sheared) sample has the highest starting value of *G*′; other starting values progressively decrease with increase of shearing temperature due to edge fracture during the 1 s of shear. The initial more rapid increase in *G*′ after shearing at 240 °C is likely from the edge-fractured sample partially rebonding to the plates.

**Figure 6:**
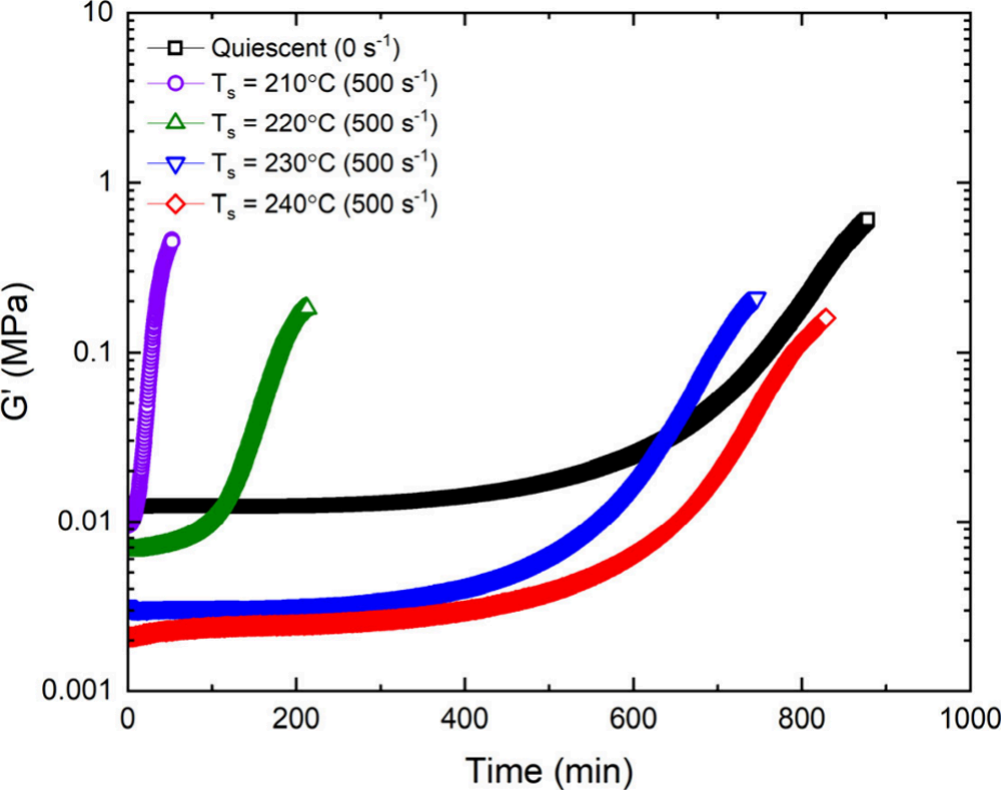
(a) Time sweep evolution at a crystallization temperature of 150 °C, where the storage modulus is monitored at 0.5 rad/s. Prior to initiating the time sweeps, samples were sheared at a perimeter shear rate of 500 s^–1^ for 1 s at their respective shearing temperatures (see legend) and cooled to 150 °C using a cooling rate of ∼10 °C/min. In the case of quiescent (no flow) crystallization, samples are cooled directly from 220 °C to the crystallization temperature of 150 °C.

Figure 7 shows the acceleration of crystallization kinetics on cooling after being sheared at various temperatures, which can be monitored through linear viscoelastic response using the Pogodina and Winter method.^45,46^ As samples crystallize, the material properties are transformed from viscous to elastic response. In shear flow, this can be monitored through the loss tangent, tan δ = *G*″(ω_0_)/*G*′(ω_0_), at constant frequency (ω_0_ = 0.5rad/sec), where *G*′ is the storage modulus and *G*′′ is the loss modulus. The crossover of the storage and loss moduli results in a value of tan δ = 1, as *G*′ surpasses *G*′′ and is used as a measure to probe changes in crystallization kinetics.

**Figure 7:**
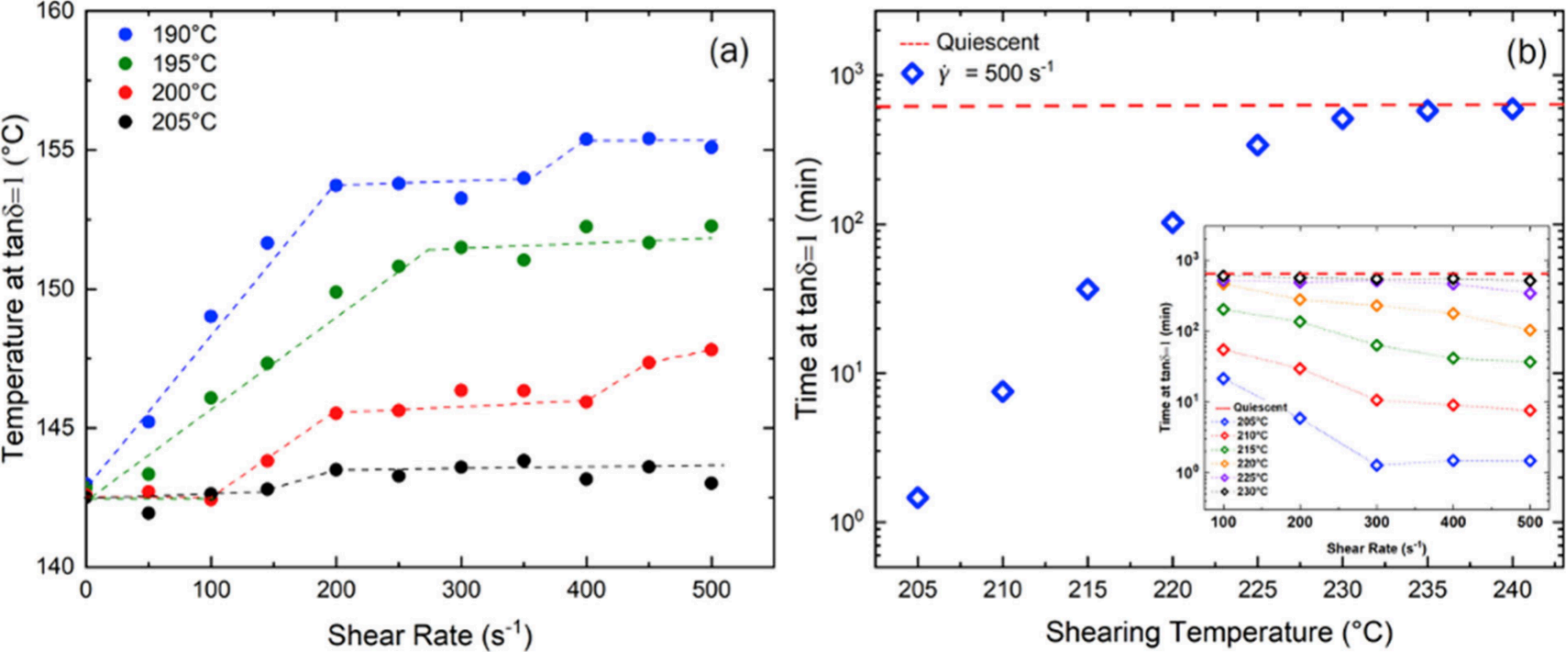
Crystallization monitored through linear viscoelasticity using a frequency of 0.5 rad/s and a strain of 0.05. Samples are sheared for 1 s using 8 mm parallel plates with a constant gap height of 0.9 mm. (a) Crystallization temperature as a function of perimeter shear rate for various shearing temperatures using a cooling rate of 3 °C/min. (b) Crystallization time at *T*_c_ = 150 °C as a function of shearing temperature. Samples are sheared at γ̇_perimeter_ = 500 s^–1^ and then quenched to a crystallization temperature of *T*_c_ = 150 °C using a cooling rate of ∼10 °C/min. The red dashed line represents the quiescent (no flow) crystallization time. The inset plot shows the kinetic development as a function of perimeter shear rate at each shearing temperature. As perimeter shear rate increases, the critical shear rate for flow effects on crystallization moves inward, lowering the crystallization time. Eventually (i.e., 300 s^–1^ at 205 °C) the critical shear rate is close enough to *r* = 0 that flow effects saturate.

At shearing temperatures *slightly* above *T*_m_° (*T*_s_ = 190–205 °C), an oscillatory temperature ramp is used to measure the temperature at which tan δ = 1, using a cooling rate of 3 °C/min (see Figure 7a). Even at such high temperatures and low undercoolings, these flow-induced precursors are robust to crystallization and do not relax back into the melt state. It could be suggested that upon the application of flow, stretched polymer chains adsorb onto fragmented particle impurities from polymerization, restricting chain mobility, and stabilizing stretched long chains that promote nucleation.^31,47,48^ At the shearing temperatures of 190, 195, and 200 °C, the crystallization temperature raises substantially with increasing deformation rates, an effect that significantly weakens when sheared at 205 °C.

In using a shearing and quenching protocol, the cooling rate is increased to ∼10 °C/min, which improves stability and reduces precursor dissolution. In Figure 7b, samples are sheared at their respective shearing temperatures at γ̇_perimeter_ = 500 s^–1^ for 1 s, and then quenched to a crystallization temperature of *T*_c_ = 150 °C to monitor isothermal crystallization kinetics as a function of time. In the case of quiescent crystallization (red dashed line) no shear is applied, and samples are cooled directly from 220 °C to *T*_c_ = 150 °C.

The crystallization kinetics are progressively reduced with elevated shearing temperatures. Interestingly, strong flow-induced precursors persist with shearing temperatures as high as *T*_s_ = 225 °C, and gradually weaken, approaching quiescent (no flow) conditions for *T*_s_ ≥ 230 °C. The inset plot of Figure 7b shows the kinetic development as a function of perimeter shear rate. With increasing levels of deformation, a greater fraction of chains can be stretched by flow, allowing for an acceleration in crystallization kinetics.

Following the method developed by Winter and Pogodina,^46^ the time-dependence of linear viscoelastic data can be normalized such that

$$G_{\mathrm{norm}}^{\prime }\,=\frac{\log[G\prime (t)/G_{\min}^{\prime }]}{\log[G_{\max}^{\prime }/G_{\min}^{\prime }]}$$

where *G*′(*t*) is the storage modulus at intermediate times, *G*′_min_ is storage modulus prior to crystallization (which is lower for samples sheared at higher temperatures from edge fracture), while *G*′_max_ is approximated to be one-third (500 MPa) the reported Young’s Modulus of semicrystalline polypropylene (∼1.5 GPa),^49^ as seen in Figure 8. The onset of crystallization is accelerated with lower shearing temperatures, and converges toward quiescent-like conditions for shearing temperatures above 230 °C.

**Figure 8:**
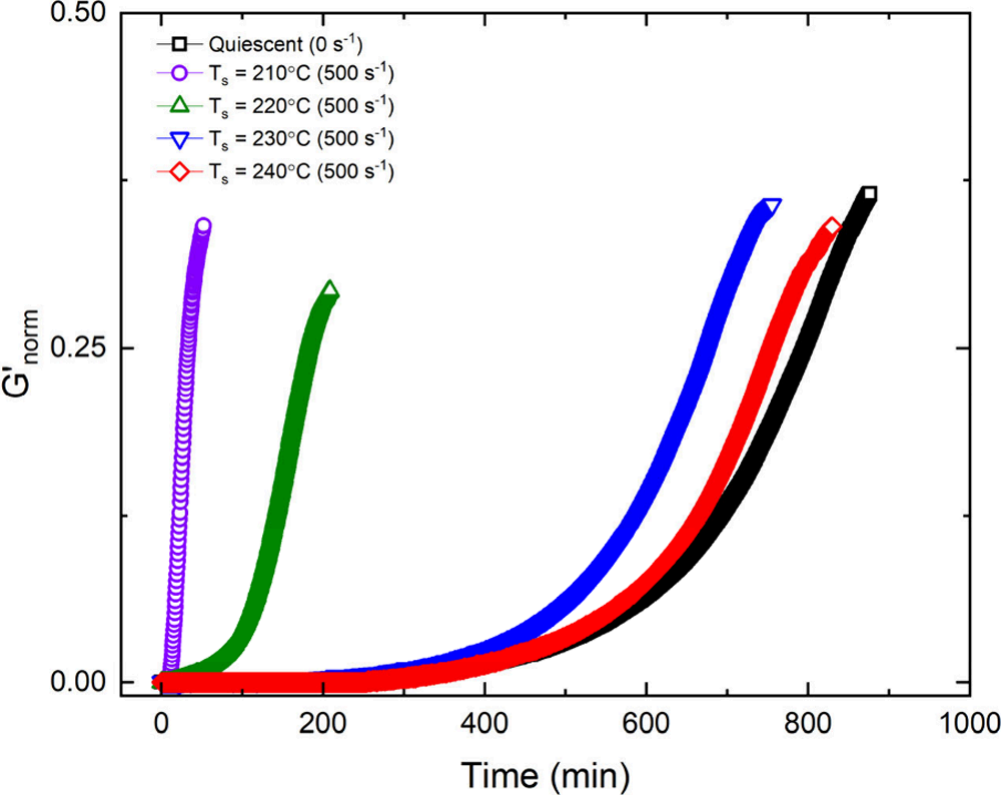
Normalized time sweep evolution of storage modulus at a crystallization temperature of 150 °C. Samples are sheared using γ̇_perimeter_ = 500 s^–1^ at elevated shearing temperatures (see legend) and cooled to the crystallization temperature at a rate of ∼10 °C/min. The storage modulus is monitored at 0.5 rad/s with a strain of 0.05 using 8 mm parallel plates with a gap height of 0.9 mm. At temperatures when anisotropic structures cease to form (≥230 °C), the rate of crystallization is drastically reduced.

The rise of the storage moduli during crystallization is due to the development of interconnected networks (gelation^45,46^ ) as crystals grow from the melt, as seen in Figures 6 and 8. Upon shearing the melt, the curves of *G*′(*t*) shift to shorter time scales, and two distinct regimes are observed. At the shearing temperatures of 210 and 220 °C, the onset of crystallization is rapid, while at *T*_s_ ≥ 230 °C, the moduli curves approach quiescent crystallization. Although anisotropic structures are unable to form at *T*_s_ = 230 °C, deformation at these high temperatures still allows for chains to be stretched and form flow-induced precursors, generating a weak nucleation effect (compare blue and black curves in Figure 8). Upon the formation of shish-kebab structures at 220 °C, the onset of crystallization is accelerated by a factor of ∼5× relative to samples sheared at 230 °C, where the morphology remains isotropic (see Figure 2). It is apparent that even weakly oriented structures play a vital role in accelerating flow-induced crystallization kinetics.

## Conclusion

In conclusion, we have shown that applying large deformations in 1 s of shear above the equilibrium melting temperature of isotactic polypropylene, it is possible to generate stable flow-induced precursors that accelerate nucleation on cooling. Anisotropic precursors could be formed with shearing temperatures as high as 220 °C (>30 °C above *T*_m_°), but were no longer metastable at or exceeding shearing temperatures of 230 °C. Near the equilibrium melting temperature, flow-induced precursors were found to be robust, even at low undercoolings, and weakened as shearing temperature is increased. From rheology, the crystallization kinetics could be accelerated even at higher shearing temperatures (up to 240 °C), as a small fraction of polymer chains could still be stretched to form flow-induced precursors with large levels of deformation.

At first glance, these results appear to be consistent with Marand’s proposed higher equilibrium melting temperature for iPP (*T*_m_° ∼ 212 °C).^17^ However, a study of the extended stability of precursors to annealing at elevated temperatures showed a strong temperature dependence in the 210–250 °C range, consistent with an activation energy of 122 kJ/mol for the precursors to disappear, three times higher than the flow activation energy of 40 kJ/mol.^48^ The sole hypothesis that seems to explain these two results is that stretched long chains adsorb to particulate impurities,^31^ such that the increase in activation energy is from requiring the precursor to desorb from the substrate.
